# Preparation of a self‐supported zeolite glass composite membrane for CO_2_/CH_4_ separation

**DOI:** 10.1002/smo.20240009

**Published:** 2024-07-11

**Authors:** Dudu Li, Mao Ye, Chao Ma, Ning Li, Zhenjie Gu, Zhihua Qiao

**Affiliations:** ^1^ State Key Laboratory of Separation Membranes and Membrane Processes Tiangong University Tianjin China; ^2^ School of Material Science and Engineering Tiangong University Tianjin China; ^3^ School of Textile Science and Engineering Tiangong University Tianjin China; ^4^ School of Physical Science and Technology Tiangong University Tianjin China; ^5^ School of Chemical Engineering and Technology Tiangong University Tianjin China

**Keywords:** CO_2_ adsorption, gas separation, stability, zeolite glass composite membrane

## Abstract

The low porosity of metal‐organic framework glass makes it difficult to prepare membranes with high permeability. To solve this problem, we fabricated a series of self‐supported zeolite glass composite membranes with different 4A zeolite loadings using the abundant pore structure of the zeolite. The 4A zeolite embedded in the zeolite glass composite membrane preserved the ligand bonds and chemical structure. The self‐supported zeolite glass composite membranes exhibited good interfacial compatibility. More importantly, the incorporation of the 4A zeolite significantly improved the CO_2_ adsorption capacity of the pure a_g_ZIF‐62 membranes. In addition, gas separation performance measurements showed that the (a_g_ZIF‐62)_0.7_(4A)_0.3_ membrane had a permeability of 13,329 Barrer for pure CO_2_ and an ideal selectivity of 31.7 for CO_2_/CH_4_, which exceeded Robeson's upper bound. The (a_g_ZIF‐62)_0.7_(4A)_0.3_ membrane exhibited good operational stability in the variable pressure test and 48 h long‐term continuous test. This study provides a method for preparing zeolite glass composite membranes.

## Graphical abstract

We have successfully prepared a self‐supported zeolite glass composite membrane, which exhibits promising CO_2_ permeability and good CO_2_/CH_4_ selectivity.

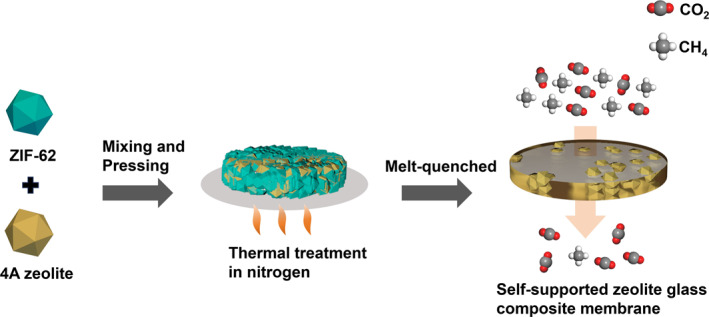

## 1 INTRODUCTION

With the advancement of industrial technology, the excessive utilization of chemical fuels has resulted in a progressive rise in CO_2_ emissions, exacerbating the greenhouse effect and causing significant detrimental effects on both society and the economy.[^1^, ^2^] Consequently, CO_2_ capture plays a pivotal role in mitigating the greenhouse effect, enhancing energy efficiency, and facilitating the efficient recycling of carbon resources. Traditional CO_2_ separation technologies encompass variable pressure adsorption,^[3]^ deep cooling separation ^[4]^ and ammonia absorption,^[5]^ but there are shortcomings such as high cost and large footprint.^[6]^ Compared with traditional separation technology, membrane separation has the characteristics of high efficiency, low energy consumption, low secondary pollution, and easy operation.[^7^, ^8^, ^9^, ^10^] Currently, membranes used for CO_2_ separation can be divided into organic membranes,[^11^, ^12^, ^13^] inorganic,[^14^, ^15^, ^16^] and hybrid membranes.[^17^, ^18^] Inorganic membranes include dense membranes and porous membranes, which are mainly prepared from inorganic materials such as zeolite, metal oxides, metal‐organic frameworks (MOFs) and carbon zeolite. Compared to organic and hybrid membranes, inorganic membranes can achieve precise screening of different components and exhibit high permeability.

In recent years, MOFs, which are open porous materials with a periodic network structure and permanent porosity composed of metal ions or ion clusters coordinated with organic ligands, have made significant progress in the field of gas separation owing to their highly tunable pore size, high porosity, high stability, and excellent reproducibility.[^19^, ^20^, ^21^, ^22^, ^23^, ^24^] MOFs are widely used in gas storage,[^25^, ^26^] separation,[^27^, ^28^] and catalysis,^[29]^ particularly in the membrane separation of CO_2_. Zeolite imidazolate frameworks (ZIFs), one subclass of the MOFs, are a class of typical nanoporous materials formed by the connection of tetrahedral metal ions (e.g. Co^2+^, Zn^2+^, Li^+^, B^3+^, and Mg^2+^) with imidazole derived ligands.[^30^, ^31^] Zeolite imidazolate frameworks have the same topology as inorganic zeolites because the ligand bonding angle of M–Im–M (M=Zn^2+^) in ZIFs materials is similar to the Si–O–Si bonding angle in inorganic zeolite (∼145°).[^32^, ^33^] However, compared to traditional inorganic zeolite, the main advantage of ZIF materials is that their pore size, skeleton chemistry, surface area, and pore volume can be precisely controlled. In addition, ZIFs have excellent chemical stability and high thermal stability and can maintain their skeleton integrity under acidic and alkaline conditions as well as high temperatures (300–500°C).[^30^, ^34^] This has the potential for application in industrial separation systems.

Several ZIFs have been found to undergo melting (e.g. ZIF‐4, ZIF‐62), forming highly viscous liquids at high temperatures that can be quenched to form glasses.[^35^, ^36^, ^37^, ^38^] Structurally, glass does not have the same periodically ordered arrangement of atoms in the long‐range range as crystalline materials; however, in the short‐range range, it displays the same short‐range order as crystal materials, that is., an orderly arranged three‐dimensional skeleton structure consisting of organic nodes and ligands.[^39^, ^40^, ^41^] With continuous research on MOF glasses, a series of MOF glass materials have been generated. Based on their composition, MOF glasses can be divided into pure and composite MOF glass.^[42]^ Composite MOF glasses can be classified into MOF crystal‐glass composites (MOF‐CGCs), blended MOF glasses, and flux melted glasses.^[42]^ Compared to the other two composite MOF glass materials, MOF‐CGCs can maintain the structural integrity of other MOF crystalline materials that cannot be melted during the melting process, and have higher gas adsorption compared to pure MOF glasses. Hou et al. dispersed MIL‐53 in a_g_ZIF‐62 to prepare a series of MOF‐CGCs materials with different loading amounts of MIL‐53.[^43^, ^44^] The glass matrix stabilized the phase transition of the flexible MIL‐53 and significantly improved its gas adsorption capacity at room temperature. Li et al. prepared MOF‐CGCs materials by dispersing crystalline ZIF‐62 and UiO‐66 in an a_g_ZIF‐62 matrix, respectively.^[45]^ Compared to pure crystalline MOF materials, CGCs have significantly improved thermodynamic properties and chemical stability. Qiao et al. prepared self‐supported MOF‐CGC membranes by blending ZIF‐8 crystals and ZIF‐62 crystals via melt quenching.^[46]^ The CGC membranes eliminated the grain boundary defects and exhibited ultrahigh ethane permeability and good C_2_H_6_/C_2_H_4_ selectivity. MOF‐CGCs membranes are promising candidates for gas separation applications. There have been numerous reports on MOF‐CGCs; however, their application in gas separation membranes has not been widespread.

4A zeolites are mesoporous aluminosilicate crystals with a special tunnel network structure, with an ideal cell composition of Na_96_Al_96_Si_96_O_384_·216H_2_O and a structure unit (α‐cage) of Na_12_[Al_12_Si_12_O_48_]·27H_2_O.[^47^, ^48^] The effective pore size of the 4A zeolite is approximately 0.4 nm (∼4 Å), with a large specific surface area and good adsorption performance.^[49]^ In addition, 4A zeolite has excellent properties such as non‐toxicity, good thermal stability, high ion exchange capacity, and environmental friendliness, and are widely used in wastewater treatment, adsorption, and other fields.[^50^, ^51^, ^52^] Therefore, in this work, we uniformly mixed 4A zeolite with ZIF‐62 crystals and melted ZIF‐62 by heating, ultimately preparing a series of self‐supported zeolite glass composite membranes. During heating, liquid ZIF‐62 was used as the continuous phase, highly thermally stable 4A zeolite was used as disperse phase, and self‐supported zeolite glass composite membranes with better interfacial compatibility were prepared because of their greater chemical compatibility. The prepared (a_g_ZIF‐62)_0.7_(4A)_0.3_ membrane exhibited positive CO_2_ permeability and good CO_2_/CH_4_ selectivity. In addition, the (a_g_ZIF‐62)_0.7_(4A)_0.3_ membrane exhibited good stability under variable pressure and long‐term operational tests.

## 2 EXPERIMENTAL SECTION

### 2.1 Chemicals and regents

Imidazole (99.5%) and benzimidazole (96%) were purchased from Energy Chemical Co., Ltd. Nanometer zinc oxide (ZnO) (<100 nm) and zinc acetate dihydrate (Zn(OAc)_2_·2H_2_O) were purchased from Aladdin Reagent Co., Ltd. 4A zeolite, dimethylformamide (DMF) and methanol were purchased from Sinopharm Chemical Reagent Co., Ltd. All chemicals and reagents were of analytical grade and used as received without further purification.

### 2.2 Material synthesis

#### Synthesis of ZIF‐62 nanoparticles

ZIF‐62 was prepared by following the mechanochemical synthesis method previously described in the literature.^[53]^ ZnO (<100 nm) (80.2 mg), Zn(OAc)_2_·2H_2_O (2.2 mg), imidazole (119.0 mg), benzimidazole (30.0 mg) and DMF (50 μL) were added to a 10 mL stainless steel grinding jar. Then, 2 × 6 mm stainless steel grinding balls were added. The grinding jar was sealed and shaken at 30 Hz for 30 min on a mixer mill. At the end of grinding, open the grinding jar and extract the powder. The powder was washed three times with DMF and methanol, respectively. Finally, ZIF‐62 powder was dried in a vacuum oven at 150°C for 24 h.

#### Preparation of the self‐supported zeolite glass composite membrane

**FIGURE 1:**
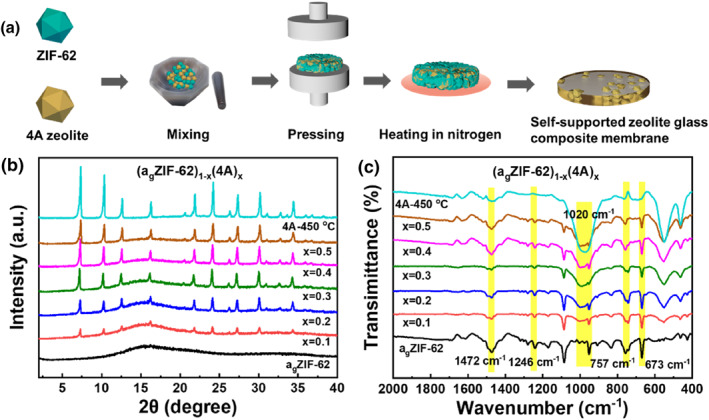
(a) Schematic diagram of the self‐supported zeolite glass composite membrane preparation. (b) XRD patterns and (c) FTIR spectra of the (a_g_ZIF‐62)_1−*x*_(4A)_*x*_ membranes. FTIR, flourier transformed infrared; XRD, x‐ray diffraction.

A series of self‐supported zeolite glass composite membranes with different mass fractions were prepared based on the preparation of MOF crystalline glass composites (MOF‐CGCs) previously reported in the literature.[^43^, ^44^, ^45^, ^46^] The 4A zeolite and ZIF‐62 powder were mixed homogeneously by grinding in a mortar for 15 min. The mass fractions (wt%) of the 4A zeolite were 0, 10, 20, 30, 40 and 50 wt%. Subsequently, 150 mg of the mixtures was pressed into 2 cm diameter pellets at 10 MPa (4 tons). The preparation of pellets prior to heating promoted close contact between the zeolite and crystals as well as the aggregation of the highly viscous liquid ZIF‐62 during heating. The pellets were then placed in a tube furnace and heated to 450°C at a rate of 3°C/min under a nitrogen atmosphere for 30 min, and then reduced to room temperature at a rate of 5^o^C/min to ultimately prepare self‐supported zeolite glass composite membrane. The pellets prior to heating and the self‐supported zeolite glass composite membranes are referred to as (ZIF‐62)_1−*x*_(4A)_*x*_ and (a_g_ZIF‐62)_1−*x*_(4A)_*x*_, respectively, where “*x*” is the mass fraction of the 4A zeolite and a_g_ indicates amorphous glass. The preparation process of the self‐supported zeolite glass composite membrane is shown in Figure 1a.

### 2.3 Material characterization

Powder x‐ray diffraction patterns acquired with Cu Kα radiation on a Bruker D8 Advance instrument were used to characterize the crystal structure of the material (2θ range from 2^o^ to 40^o^). The Flourier transformed infrared (FTIR) spectroscopy tested on the Bruker TENSOR II instrument was used to study the molecular structure and composition of materials, with the wavenumber range of 400–2000 cm^−1^. The content of the elements in the 4A zeolite was obtained by X‐ray Fluorescence Spectrometer (XRF) testing. The specific surface area of 4A zeolite was tested using the Brunauer‐Emmett‐Teller (BET) method using N_2_ isotherms at 77 K. The pore size distribution of the self‐supported zeolite glass composite membrane was obtained from CO_2_ adsorption using BSD‐660S instrument at 195 K. The morphology of the material, surface and cross‐section of the membranes were characterized using ZEISS Gemini SEM500 instrument under a 5 kV acceleration voltage. The lattice stripes of 4A zeolite and the amorphous morphology of a_g_ZIF‐62 were characterized by HR‐TEM images obtained using a JEOL JEM‐F200 instrument at 200 kV. Thermogravimetric analysis (TGA) was performed on a Netzsch STA449F3 instrument, and the samples were heated from room temperature to 800°C under nitrogen atmosphere at a rate of 10^o^C/min. Differential scanning calorimeter (DSC) curves were obtained on TA DSC 250. Under nitrogen atmosphere, the samples were first raised from room temperature to 500°C at a heating rate of 10°C/min, cooled to room temperature at a rate of 20^o^C/min, then raised to 500°C at a heating rate of 10^o^C/min and finally cooled to room temperature. The volume composition of the elements analyzed was characterized by Energy Dispersive X‐Ray Spectroscopy (EDS) spectra. The ^29^Si MAS NMR measurements were performed on the America Agilent 600M instrument. The CO_2_ and N_2_ adsorption isotherms of the self‐supported zeolite glass composite membranes were tested using a Microparticle ASAP 2020 PLUS HD88 instrument at 298 K.

### 2.4 Gas permeation measurement

The permeation and selectivity properties of the self‐supported zeolite glass composite membranes were investigated at 25°C using the constant‐pressure method. Prior to gas permeation measurements, the feed and carrier gases were dried and did not contain water. The sample was fixed in a circular stainless steel membrane cell with a sealing ring, and its effective membrane area was 0.018 cm^2^. Helium was selected as the carrier gas and the flow rate was maintained at 5 cm^3^/min. The flow rate of the feed gas was maintained at 30 cm^3^/min, and testing was performed on pure CO_2_, pure CH_4_, and a mixed gas CO_2_/CH_4_ (50/50). The trans‐membrane pressure difference of the feed gas was measured at 0.2–1.0 MPa. The gas composition in downstream of the permeation was analyzed using gas chromatography. The membranes were tested at three different locations for each parameter, and each membrane was cycled at least 10 times to ensure data accuracy. Finally, the average value and standard error (<5%) were used to evaluate the performance of the membrane. The permeability of the membrane can be obtained by the following Equation (1):

(1) $$P_{i}=\frac{Q_{i}\times L}{{∆P}_{i}\times S}$$

The ideal selectivity of the membrane can be obtained by the following Equation (2):

(2) $${\;\alpha }_{i/j}=\frac{P_{i}}{P_{j}}$$

The separation factor of mixed gases containing two gas components can be obtained by the following Equation (3)

(3) $$\alpha_{i/j}=\frac{y_{i}/y_{j}}{x_{i}/x_{j}}$$

where *P*_*i*_ is the gas permeability (Barrer, 1 Barrer = 10^−10^ cm^3^ (STP) cm cm^−2^ s cmHg^−1^), *Q*_*i*_ is the permeate flow rate of the gas (cm^3^(STP)s^−1^), *L* is the thickness of the membrane (cm), ∆*P*_*i*_ is the trans‐membrane pressure difference (cmHg), *S* is the effective area of the membrane (cm^2^), *α* is the separation factor, *x* and *j* are the percentages of gas components in the feed gas, respectively.

## 3 RESULTS AND DISCUSSION

### 3.1 The characterization of (ZIF‐62)_1−*x*_(4A)_*x*_ and (a_g_ZIF‐62)_1−*x*_(4A)_*x*_ membrane

ZIF‐62 crystals were successfully prepared according to a previously reported method (see material synthesis,^[53]^) and the morphologies of the ZIF‐62 crystals and 4A zeolite were investigated (Figure S1). The elemental content of Al and Si in the 4A zeolite particles were determined using XRF, and the results showed that the elemental content ratio of Si:Al was approximately 1.3 (Table S1). Subsequently, a series of mixtures (ZIF‐62)_1−*x*_(4A)_*x*_ with different mass fractions were prepared by uniformly mixing the 4A zeolite and ZIF‐62 crystal in a mortar. When the mass fraction of 4A zeolite is 0 wt%, the peaks of ZIF‐62 crystal at 2θ values of 11.1º, 12.5º, 13.4º and 16.8º correspond to the crystal lattice planes (110), (021), (111) and (101), respectively (Figure S2a),^[54]^ consistent with the ZIF‐62 crystal structure reported previously.[^55^, ^56^] The mixtures were then pressed into pellets and heated in a tube furnace to obtain (a_g_ZIF‐62)_1−*x*_(4A)_*x*_ membranes. The XRD patterns of the (a_g_ZIF‐62)_1−*x*_(4A)_*x*_ membranes are shown in Figure 1b, and the results indicate that when the mass fraction of 4A zeolite is 0 wt%, the ZIF‐62 glass has no obvious XRD peaks, which confirms the role of ZIF‐62 vitrification. As the mass fraction of the 4A zeolite increased, the Bragg diffraction peaks of the 4A zeolite gradually strengthened. This is because liquid ZIF‐62 is not sufficient to seal the 4A zeolite, resulting in the retention of its crystal structure.

The chemical compositions of the (ZIF‐62)_1−*x*_(4A)_*x*_ and (a_g_ZIF‐62)_1−*x*_(4A)_*x*_ membranes were investigated using FTIR spectroscopy. As shown in Figure 1c and Figure S2b, the peaks at 1246 and 673 cm^−1^ are caused by the stretching vibration of the Zn–N bond and the C–H bond in imidazole, respectively,^[54]^ the peaks at 757 and 1472 cm^−1^ are caused by benzene,^[57]^ and the peak at 1020 cm^−1^ is caused by the stretching vibration of the Si–O–Si bond in the zeolite of 4A,^[58]^ indicating that the functional groups and chemical bonds of the self‐supported zeolite glass composite membranes were not decomposed.

**FIGURE 2:**
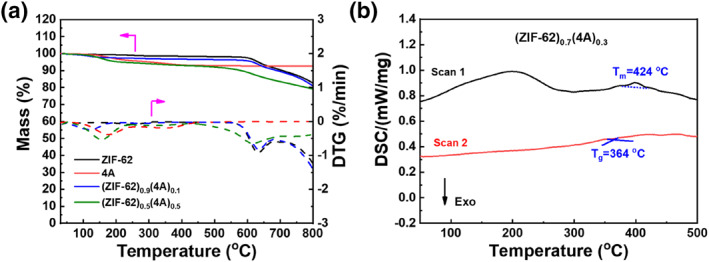
(a) TGA curves of the (ZIF‐62)_1−*x*_(4A)_*x*_. Dashed lines indicate the derivative of the weight loss versus temperature. (b) DSC curves of the (ZIF‐62)_0.7_(4A)_0.3_. The exothermic (EXO) direction is downward. DSC, differential scanning calorimeter; TGA, thermogravimetric analysis.

**FIGURE 3:**
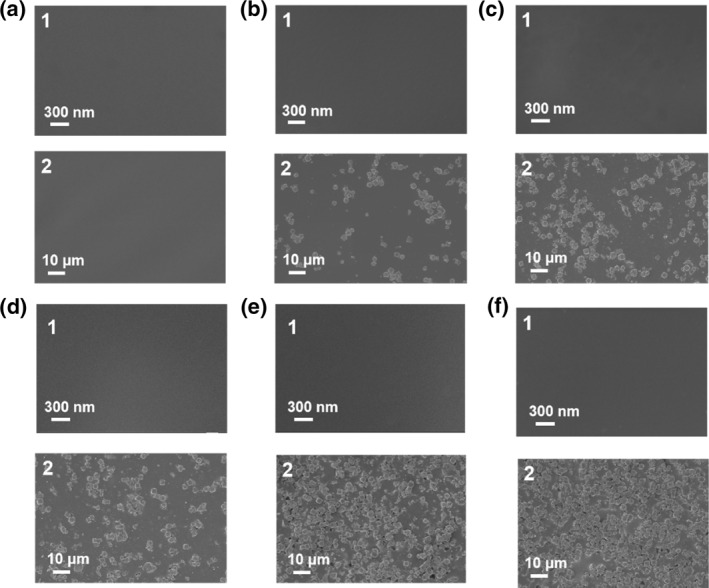
SEM images of the self‐supported zeolite glass composite membranes. (a) a_g_ZIF‐62, (b) (a_g_ZIF‐62)_0.9_(4A)_0.1_, (c) (a_g_ZIF‐62)_0.8_(4A)_0.2_, (d) (a_g_ZIF‐62)_0.7_(4A)_0.3_, (e) (a_g_ZIF‐62)_0.6_(4A)_0.4_, (f) (a_g_ZIF‐62)_0.5_(4A)_0.5_.

The TGA curves of ZIF‐62 particles and mixture (ZIF‐62)_1−*x*_(4A)_*x*_ are shown in Figure 2a. As shown in Figure 2a, the ZIF‐62 particles and 4A zeolite did not decompose at 500°C, exhibiting good thermal stability. The decomposition of ZIF‐62 particles began at 600°C, which was attributed to the collapse of the skeleton structure due to the decomposition of organic ligands and metal sites in ZIF‐62 particles.^[54]^ The DSC curves of the ZIF‐62 particles are shown in Figure 3. The heat absorption peaks at 428 and 335°C represent the melting temperature (*T*_m_) and glass transition temperature (*T*_g_) of the ZIF‐62 particles, respectively, thus confirming the vitrification of ZF‐62. As shown in Figure 2b, the addition of 4A zeolite had almost no effect on the *T*_m_ of the ZIF‐62 particles. However, the *T*_g_ of the mixtures (ZIF‐62)_0.7_(4A)_0.3_ were higher than that of the pure ZIF‐62 particles, which was because the 4A zeolite affected the movement of the molecular chain segments inside the ZIF‐62 particles during the melt quenching process of the ZIF‐62 particles.

The morphologies of the self‐supported zeolite glass composite membranes were characterized by SEM. As shown in Figure 3, the surface of the pure ZIF‐62 glass membrane is smooth and dense without any visual defects. With the increase of 4A zeolite incorporation ratio, the membranes surface of (a_g_ZIF‐62)_0.9_(4A)_0.1_ to (a_g_ZIF‐62)_0.5_(4A)_0.5_ had no visual defects at high magnification. However, the membrane surface exhibits a rougher morphology and 4A zeolite particles can be observed at low magnification. In addition, significant grain boundary defects were found in the (a_g_ZIF‐62)_0.6_(4A)_0.4_ and (a_g_ZIF‐62)_0.5_(4A)_0.5_ membranes, which was attributed to the fact that liquid ZIF‐62 was insufficient to completely seal the 4A zeolite at high contents. Cross‐sectional SEM images (Figure S4) show that the thickness of the self‐supported zeolite glass composite membrane is within the range of 380–420 μm, confirming that the zeolite glass membranes can be self‐supported for gas separation.

**FIGURE 4:**
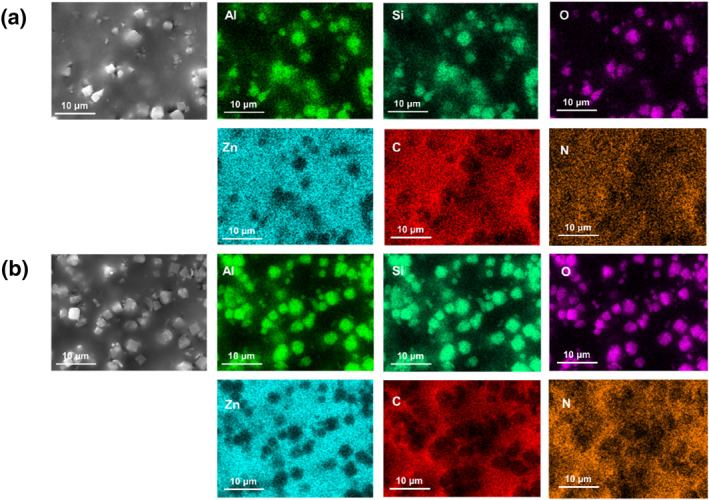
Surface element distribution of the self‐supported zeolite glass composite membranes. (a) (a_g_ZIF‐62)_0.9_(4A)_0.1_ membrane, (b) (a_g_ZIF‐62)_0.7_(4A)_0.3_ membrane.

SEM‐EDS and HR‐TEM‐EDS were used to characterize the elemental distribution of the surface metal centers. Elemental mapping of Al, Si and O confirmed the presence of 4A zeolite particles, whereas elemental mapping of Zn, C and N confirmed the presence of the ZIF‐62 glass matrix. Figure 4 shows that both membranes (a_g_ZIF‐62)_0.9_(4A)_0.1_ and (a_g_ZIF‐62)_0.7_(4A)_0.3_ exhibited relatively dense surfaces, demonstrating that the 4A zeolite were uniformly embedded in the ZIF‐62 glass matrix and were in close contact with a_g_ZIF‐62. The (a_g_ZIF‐62)_0.9_(4A)_0.1_ and (a_g_ZIF‐62)_0.7_(4A)_0.3_ membranes were ground into powder and characterized by HR‐TEM and HR‐TEM‐EDS to study the microstructure of the self‐supported zeolite glasses (Figures S5 and S6). The HR‐TEM image shows the lattice striations of the crystalline phase of the 4A zeolite in the (a_g_ZIF‐62)_0.7_(4A)_0.3_ membrane as well as the close contact between the crystalline and amorphous regions. Furthermore, selected area electron diffraction tests were conducted on the (a_g_ZIF‐62)_0.7_(4A)_0.3_ membrane, confirming the presence of 4A zeolite crystalline phase. The HR‐TEM‐EDS mapping results indicate that the 4A zeolite was uniformly distributed in the a_g_ZIF‐62 matrix, which is consistent with the results shown in Figure 4.

**FIGURE 5:**
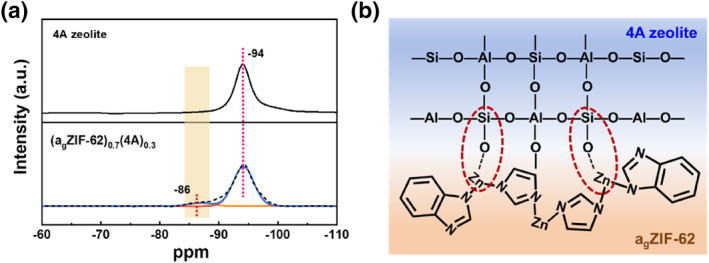
(a) ^29^Si MAS NMR spectra of 4A zeolite and (a_g_ZIF‐62)_0.7_(4A)_0.3_, (b) Scheme of the interface and interaction between 4A zeolite and a_g_ZIF‐62.

Furthermore, in order to investigate the interface between 4A zeolite and a_g_ZIF‐62, 4A zeolite and (a_g_ZIF‐62)_0.7_(4A)_0.3_ membrane using ^29^Si MS NMR were tested. As shown in Figure 5, the peak at −94 ppm for 4A zeolite is attributed to Si atoms (Si‐O‐Al).^[59]^ In addition, the (a_g_ZIF‐62)_0.7_(4A)_0.3_ membrane shows a significant peak at −86 ppm, suggesting that there is an interaction between the 4A zeolite and a_g_ZIF‐62 in the (a_g_ZIF‐62)_0.7_(4A)_0.3_ membrane, that is, the generation of Si‐O‐Zn.[^60^, ^61^] Similarly, there may be interactions between Al atoms in 4A zeolite and Zn atoms in a_g_ZIF‐62, but the ^27^Al nuclei exhibit a severe quadrupole effect under magnetic field, making it difficult to quantitatively analyze Al‐O‐Zn under MAS NMR tests.^[62]^

### 3.2 The characterization of gas separation properties of self‐supported zeolite glass composite membranes

**FIGURE 6:**
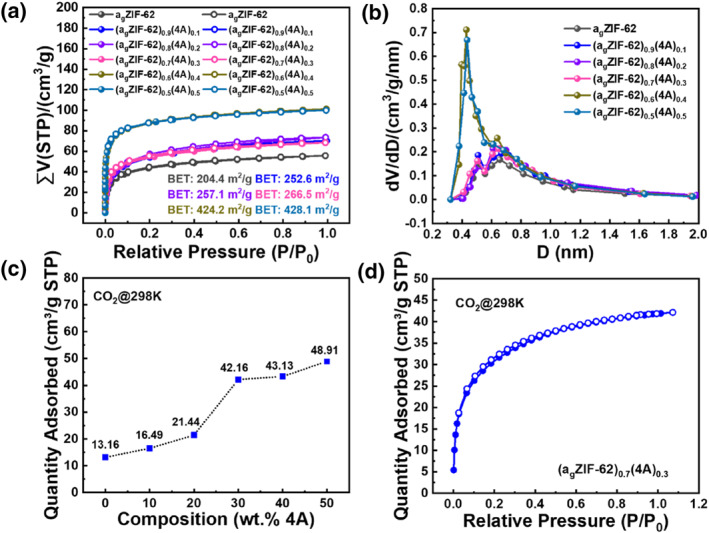
(a) CO_2_ adsorption (solid points)‒desorption (open points) isothermal curves of the (a_g_ZIF‐62)_1−*x*_(4A)_*x*_ membrane at 195 K. (b) Micropore size distribution of the (a_g_ZIF‐62)_1−*x*_(4A)_*x*_ membranes calculated by the H‐K (Original) method using CO_2_ adsorption isotherm curves. (c) Quantity adsorbed from gas adsorption isotherms for the (a_g_ZIF‐62)_1−*x*_(4A)_*x*_ series using CO_2_ gas at 298 K. (d) CO_2_ adsorption isotherm curves of the (a_g_ZIF‐62)_0.7_(4A)_0.3_ membrane.

The diffusion limitation of N_2_ molecules into the narrow pores of the 4A zeolite at low temperatures resulted in a lower adsorption capacity of N_2_ at 77 K, showing a smaller specific surface area of the 4A zeolite (Figure S7a). CO_2_ and N_2_ adsorption tests were conducted on the 4A zeolite at 298 K, and the results showed that the 4A zeolite had a high adsorption capacity for CO_2_ (Figure S7b). Therefore, to better characterize the specific surface area and pore size distribution of self‐supported zeolite glass composite membranes, CO_2_ was chosen as the adsorption gas to test the specific surface area of the self‐supported zeolite glass composite membranes. As shown in Figure 6a,b, the pure ZIF‐62 glass membrane had low porosity. With the increase of 4A zeolite incorporation rate, the specific surface area of the self‐supported zeolite glass composite membranes increased and the pore size distribution shifted toward narrower pores close to 0.4 nm, indicating that the nanopores of the 4A zeolite were retained in the self‐supported zeolite glass composite membranes, which is consistent with the XRD results. In addition, the 4A zeolite increased the porosity of the a_g_ZIF‐62 membrane.

The relationship between the 4A zeolite loading and gas uptake characteristics was explored using CO_2_ adsorption isotherms for a series of self‐supported zeolite glass composite membranes (Figure 6c,d and Figure S8). As shown in Figure 6c, the CO_2_ adsorption capacity of the pure a_g_ZIF‐62 membrane is 13.16 (cm^3^/g STP). The adsorption CO_2_ capacity for membranes (a_g_ZIF‐62)_0.9_(4A)_0.1_ to (a_g_ZIF‐62)_0.5_(4A)_0.5_ increased from 16.49 to 48.91 cm^3^/g STP. These results indicate that an increase in the incorporation ratio of 4A zeolite leads to an increase in the porosity of the self‐supported zeolite glass composite membranes.

**FIGURE 7:**
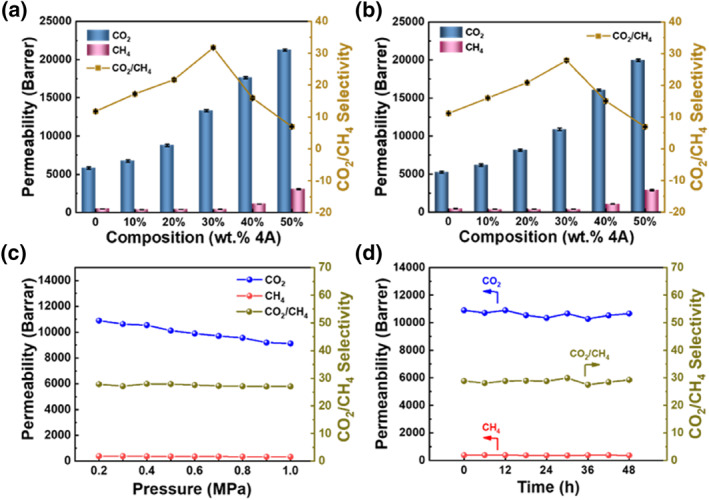
Gas separation performance of the (a_g_ZIF‐62)_1−*x*_(4A)_*x*_ membranes. (a) Pure‐gases CO_2_, CH_4_ permeance and ideal CO_2_/CH_4_ gas selectivity in self‐supported zeolite glass composite membranes. (b) Mixed‐gases (50/50) CO_2_, CH_4_ permeance and CO_2_/CH_4_ gas selectivity in self‐supported zeolite glass composite membranes. (c) Mixed‐gas (50/50) separation properties of (a_g_ZIF‐62)_0.7_(4A)_0.3_ membrane for CO_2_ and CH_4_ at different pressure. (d) Long‐term stability testing with 48 h of mixed gas (CO_2_/CH_4_ = 50/50) for (a_g_ZIF‐62)_0.7_(4A)_0.3_ membrane.

The gas permeability and separation properties of the prepared self‐supported zeolite glass composite membranes were evaluated using a constant‐pressure method. Figure 7a shows the pure gas performance of the self‐supported zeolite glass composite membranes. The CO_2_ permeability of the a_g_ZIF‐62 membrane was 5838 Barrer, and the ideal selectivity for CO_2_/CH_4_ was 11.8. With increasing incorporation of the 4A zeolite, the permeability of membranes (a_g_ZIF‐62)_0.9_(4A)_0.1_ to (a_g_ZIF‐62)_0.5_(4A)_0.5_ increased from 6758 to 21,253 Barrer. In addition, the ideal selectivity of membranes (a_g_ZIF‐62)_0.9_(4A)_0.1_ to (a_g_ZIF‐62)_0.7_(4A)_0.3_ increased from 17.2 to 31.7. Conversely, the ideal selectivity of membranes (a_g_ZIF‐62)_0.6_(4A)_0.4_ and (a_g_ZIF‐62)_0.5_(4A)_0.5_ decreased because liquid ZIF‐62 was not sufficient to completely seal the high content of 4A zeolite. This resulted in defects in membranes (a_g_ZIF‐62)_0.6_(4A)_0.4_ and (a_g_ZIF‐62)_0.5_(4A)_0.5_, which made the selectivity decrease. In contrast, the (a_g_ZIF‐62)_0.7_(4A)_0.3_ membrane exhibited good gas separation performance. The CO_2_ permeability of the (a_g_ZIF‐62)_0.7_(4A)_0.3_ membrane was 13,329 Barrer and the ideal selectivity for CO_2_/CH_4_ was 31.7. The self‐supported zeolite glass composite membranes were further tested using a binary gas mixture (CO_2_/CH_4_) (Figure 7b). The CO_2_ permeability of the (a_g_ZIF‐62)_0.7_(4A)_0.3_ membrane was 10,896 Barrer, and the selectivity of the CO_2_/CH_4_ (50/50) mixture was 27.9. The permeability of the mixed gases was lower than that of the pure gases, which was attributed to the competing adsorption of gases.

The gas separation performance of the (a_g_ZIF‐62)_0.7_(4A)_0.3_ membrane at different pressures was investigated. Figure 7c shows the effect of pressure on the mixed gas separation performance for CO_2_/CH_4_. Notably, the permeability of CO_2_ decreased from 10,896 to 9120 Barrer as the feed pressure increased from 0.2 to 1.0 MPa, whereas the permeability of CH_4_ remained essentially unchanged. Thus, the selectivity for the mixed gas CO_2_/CH_4_ decreased slightly. The long‐term stability of the (a_g_ZIF‐62)_0.7_(4A)_0.3_ membrane for mixed gas CO_2_/CH_4_ was further investigated (Figure 7d). The (a_g_ZIF‐62)_0.7_(4A)_0.3_ membrane had good long‐term stability, and the permeability of CO_2_ and CH_4_ as well as the gas separation performance of CO_2_/CH_4_ remained unchanged during the 48‐h test.

**FIGURE 8:**
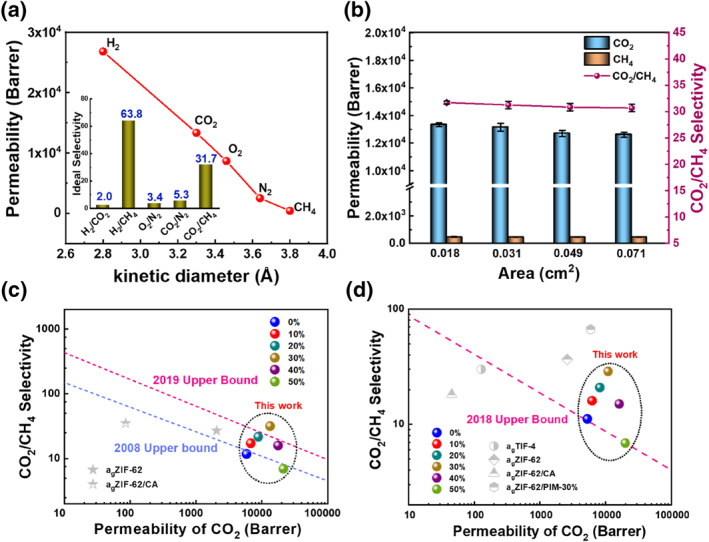
(a) Gas separation performance of the (a_g_ZIF‐62)_0.7_(4A)_0.3_ membrane for five different gases. Effective testing area: 0.018 cm^2^. (b) The pure CO_2_, CH_4_ permeability and ideal CO_2_/CH_4_ selectivity of the (a_g_ZIF‐62)_0.7_(4A)_0.3_ membrane with different test areas. (c) Comparison of pure gas separation performance of the (a_g_ZIF‐62)_1−*x*_(4A)_*x*_ membranes with those of other reported membranes. (d) Comparison of mixed gas separation performance of the (a_g_ZIF‐62)_1−*x*_(4A)_*x*_ membranes with those of other reported membranes. The 2008, 2019 pure‐gas and 2018 mixed‐gas for CO_2_/CH_4_ Upper Bound lines are from references of,[^63^, ^64^, ^65^] respectively.

The 4A zeolite plays a very important role in self‐supported zeolite glass composite membranes, which is mainly attributed to the following reasons. First, the 4A zeolite, with its high thermal stability and pore size of 4 Å,^[49]^ has a greater adsorption capacity for CO_2_ (3.3 Å) than that for CH_4_ (3.8 Å), making it possible to separate CO_2_ and CH_4_. Second, during the melting and quenching of ZIF‐62, the crystalline structure of the 4A zeolite is retained, thus improving the CO_2_ adsorption capacity of the pure ZIF‐62 glass membrane. Third, the incorporation of 4A zeolite improves the porosity of the pure ZIF‐62 glass membrane. These reasons may explain the potential of the self‐supported zeolite glass composite membranes for CO_2_/CH_4_ separation. The effects of different gases and test areas on the gas separation performance of (a_g_ZIF‐62)_0.7_(4A)_0.3_ membranes were investigated. As shown in Figure 8a, membrane (a_g_ZIF‐62)_0.7_(4A)_0.3_ exhibits high permeability to H_2_ due to its smallest kinetic diameter and an ideal selectivity of 63.8 for H_2_/CH_4_. Figure 8b shows that different test areas have a little effect on the gas separation performance of the (a_g_ZIF‐62)_0.7_(4A)_0.3_ membrane, indicating that the performance of the (a_g_ZIF‐62)_0.7_(4A)_0.3_ membrane is basically stable. The separation performance of the (a_g_ZIF‐62)_0.7_(4A)_0.3_ membrane was compared to that of CO_2_/CH_4_ reported in the literature (Figure 8c,d and Table S2‐3). The ideal selectivity for CO_2_/CH_4_ exceeded Robeson's upper bound (2019), whereas the selectivity for the mixed gas CO_2_/CH_4_ exceeded that of Robeson's upper bound (2018). The (a_g_ZIF‐62)_0.7_(4A)_0.3_ membrane exhibited good CO_2_ permeability and CO_2_/CH_4_ selectivity, making it a potential candidate material for the separation of CO_2_/CH_4_.

## 4 CONCLUSION

In summary, a series of self‐supported zeolite glass composite membranes (a_g_ZIF‐62)_1−*x*_(4A)_*x*_ were successfully prepared by dispersing highly thermally stable 4A zeolite in ZIF‐62 and quenching ZIF‐62 by melting. The self‐supported zeolite glass composite membranes exhibited good interfacial compatibility and retained the nanostructure of the 4A zeolite, which significantly improved their CO_2_ adsorption capacity at room temperature. The self‐supported zeolite glass composite membrane (a_g_ZIF‐62)_0.7_(4A)_0.3_ exhibited good gas separation performance when the mass fraction of the 4A zeolite incorporation was 30 wt%. Compared to the pure a_g_ZIF‐62 membrane, the pure gas CO_2_ permeability and ideal CO_2_/CH_4_ selectivity of the (a_g_ZIF‐62)_0.7_(4A)_0.3_ membrane increased by 128% and 169%, respectively. The mixed gas test results showed that CO_2_ permeability and CO_2_/CH_4_ selectivity of the (a_g_ZIF‐62)_0.7_(4A)_0.3_ membrane increased by 108% and 159%, respectively. Additionally, the (a_g_ZIF‐62)_0.7_(4A)_0.3_ membrane exhibited good stability. The gas separation performance of the (a_g_ZIF‐62)_0.7_(4A)_0.3_ membrane remained essentially unchanged after different pressure tests and continuous testing for 48 h. This strategy provides a method for preparing self‐supported zeolite glass composite membranes.

## AUTHOR CONTRIBUTIONS

**Dudu Li**: Conceptualization; methodology; investigation; data curation; writing original draft. **Mao Ye**: Methodology; validation; investigation; data curation. **Chao Ma:** Investigation; writing reviewing. **Ning Li**: Methodology; validation. **Zhenjie Gu:** Resources; validation; project administration; supervision. **Zhihua Qiao**: Resources; validation; supervision; conceptualization.

## CONFLICT OF INTEREST STATEMENT

The author declares that there is no conflict of interest.

## ETHICS STATEMENT

The authors declare that there are no ethical issues involved.

## Supporting information

Supporting Information S1

## Data Availability

Data are available on request from the authors. The data that support the findings of this study are available from the corresponding author upon reasonable request.
