# Supplementary material for: Preparation of a self‐supported zeolite glass composite membrane for CO2/CH4 separation
Source: Smart Mol. 2024 Jul 11;2(3):e20240009. doi: 10.1002/smo.20240009 (PMC12118213; doi:10.1002/smo.20240009)
Supplement: Supplementary file 1 — Supporting Information S1 [file SMO2-2-e20240009-s001.docx]

**Preparation of a self-supported zeolite glass composite membrane for CO_2_/CH_4_ separation**

Dudu Li ^a, b^, Mao Ye ^a, c^, Chao Ma ^a, b^, Ning Li ^a, b^, Zhenjie Gu * ^a, d^, Zhihua Qiao * ^a, e^

a. State Key Laboratory of Separation Membranes and Membrane Processes, Tiangong University, Tianjin 300387, China.

b. School of Material Science and Engineering, Tiangong University, Tianjin 300387, China.

c. School of Textile Science and Engineering, Tiangong University, Tianjin, 300387, China.

d. School of Physical Science and Technology, Tiangong University, Tianjin, 300387, China.

e. School of Chemical Engineering and Technology, Tiangong University, Tianjin 300387, China.

*Corresponding authors: Prof. Zhihua Qiao, Zhenjie Gu.

E-mail: [qiaozhihua@tiangong.edu.cn](mailto:qiaozhihua@tiangong.edu.cn), gzjtgd@163.com.

**Results and discussion**


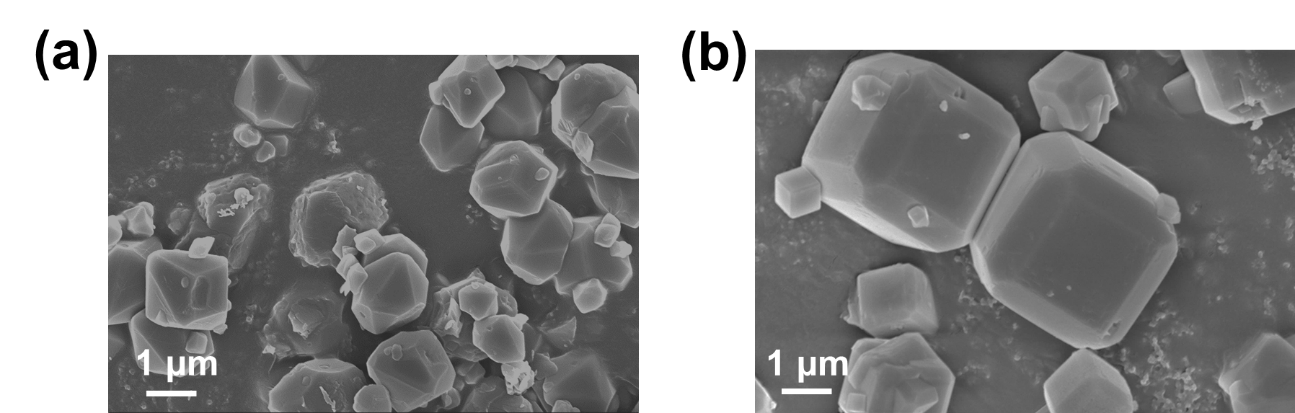


**Fig. S1** SEM images of (a) ZIF-62 and (b) 4A zeolite.


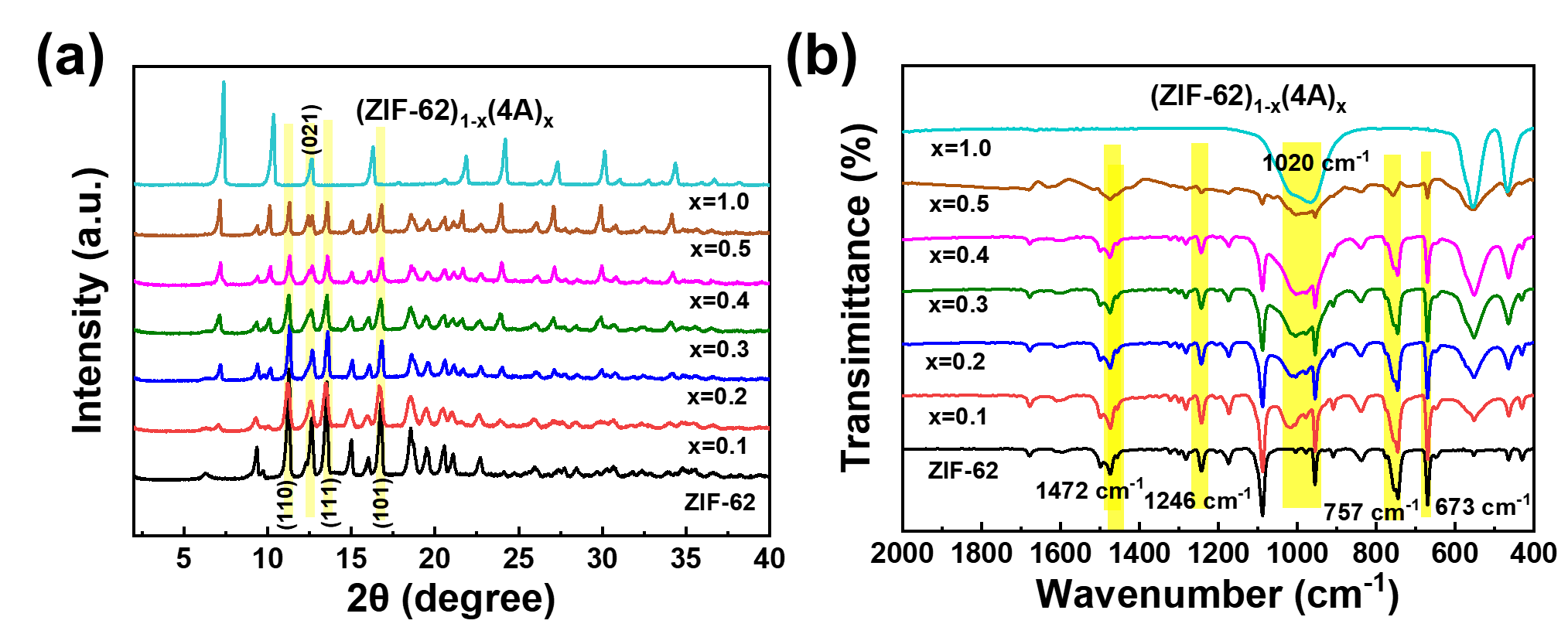


**Fig. S2** (a) XRD patterns and (b) FTIR spectra of (ZIF-62)_1-x_(4A)_x_.

**
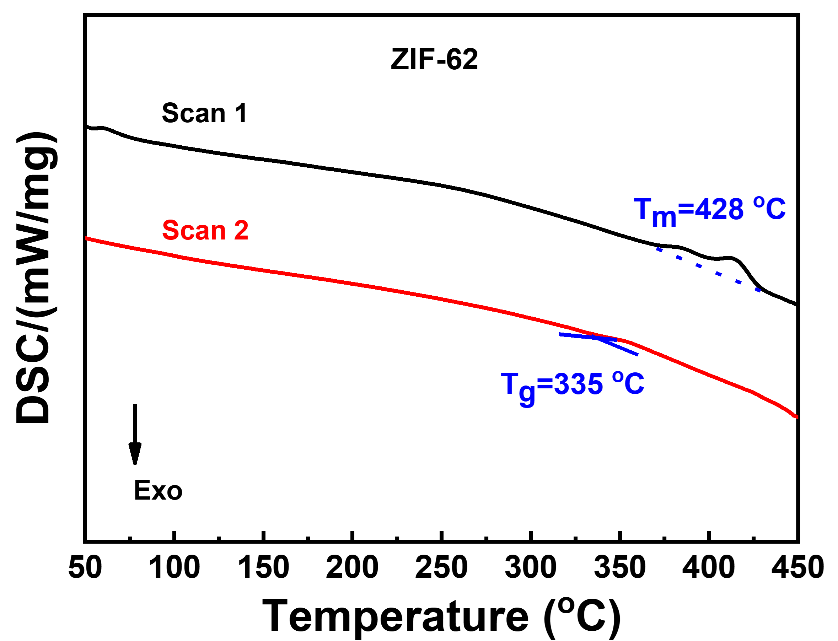
**

**Fig. S3** DSC curves of ZIF-62. The exothermic (EXO) direction is downward.

**
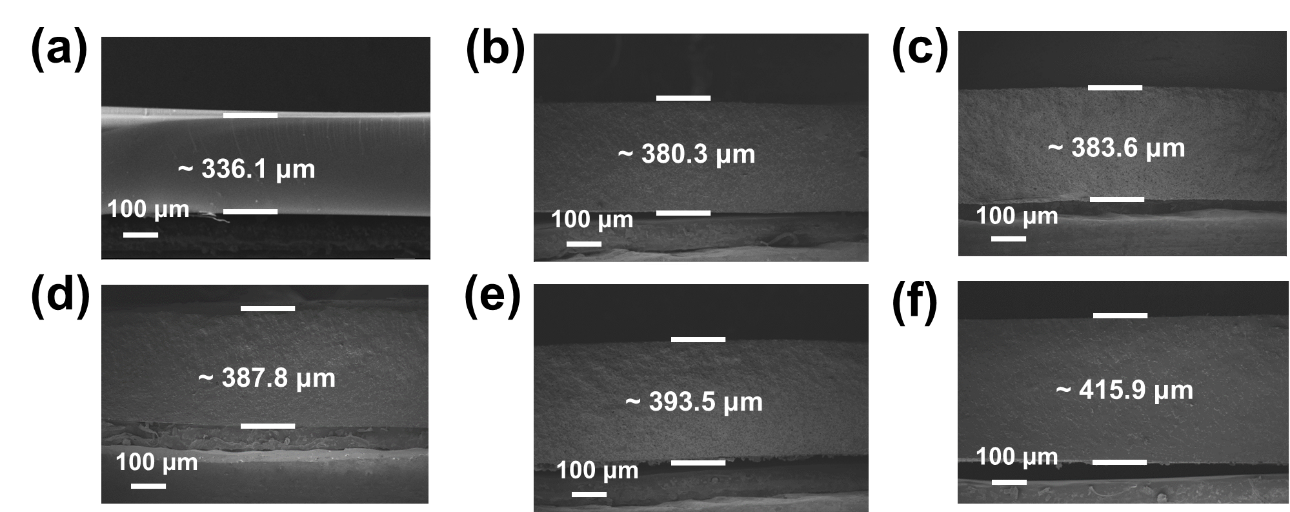
**

**Fig. S4** SEM cross-sectional images of the self-supported zeolite glass composite membranes. (a) a_g_ZIF-62, (b) (a_g_ZIF-62)_0.9_(4A)_0.1_, (c) (a_g_ZIF-62)_0.8_(4A)_0.2_, (d) (a_g_ZIF-62)_0.7_(4A)_0.3_, (e) (a_g_ZIF-62)_0.6_(4A)_0.4_, (f) (a_g_ZIF-62)_0.5_(4A)_0.5_.

**
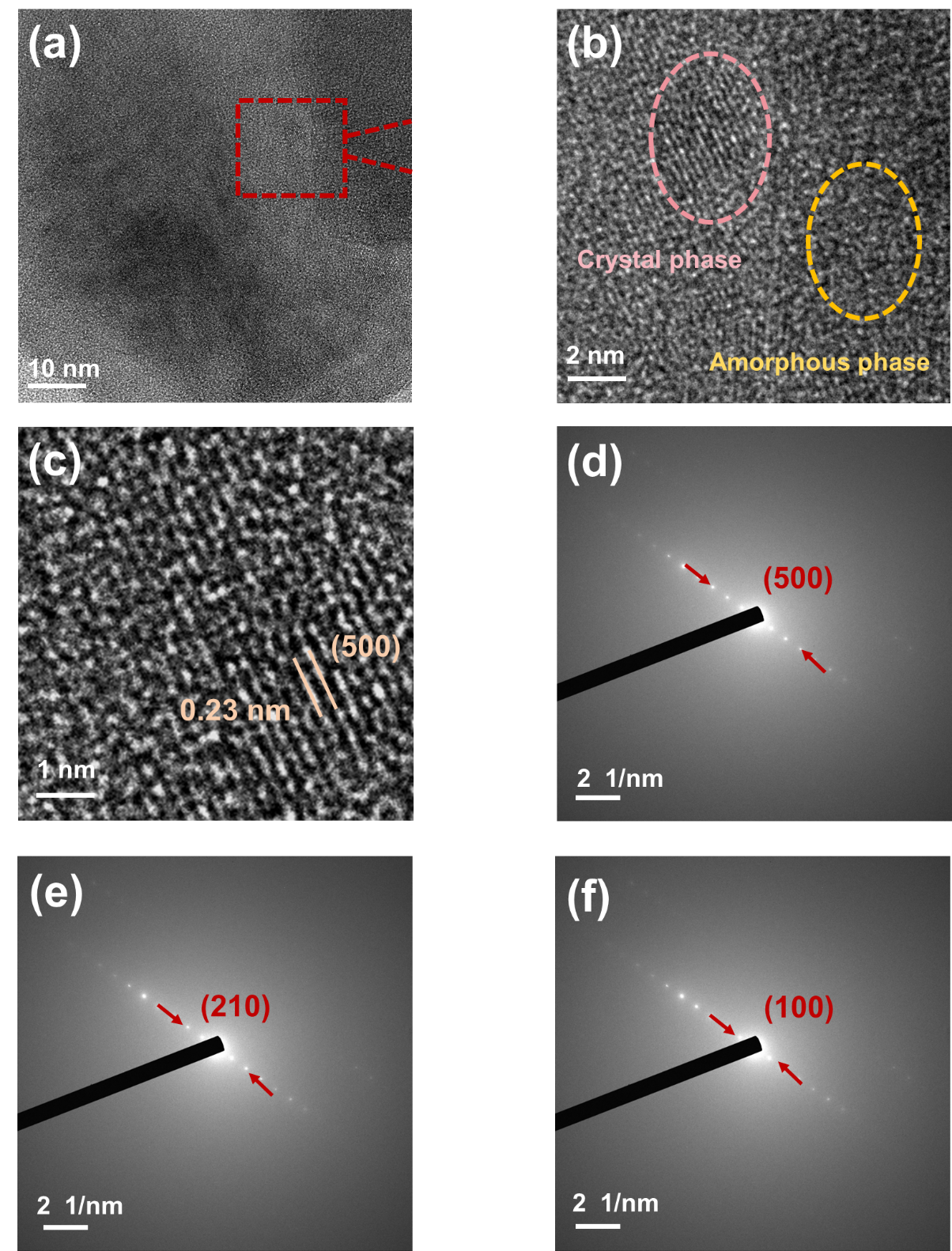
**

**Fig. S5** HR-TEM images of (a_g_ZIF-62)_0.7_(4A)_0.3_. (a) HR-TEM images, (b) Enlarged HR-TEM image, (c) Enlarged HR-TEM image of the crystal phase, and the crystal plane spacing *d* = 0.23 nm, corresponding to (500) spacing for 4A zeolite. (d-f) Selected area electron diffraction (SAED) patterns of the 4A zeolite. The points indicated by the red arrows correspond to (500), (210), and (110) spacing of 4A zeolite, respectively.

**
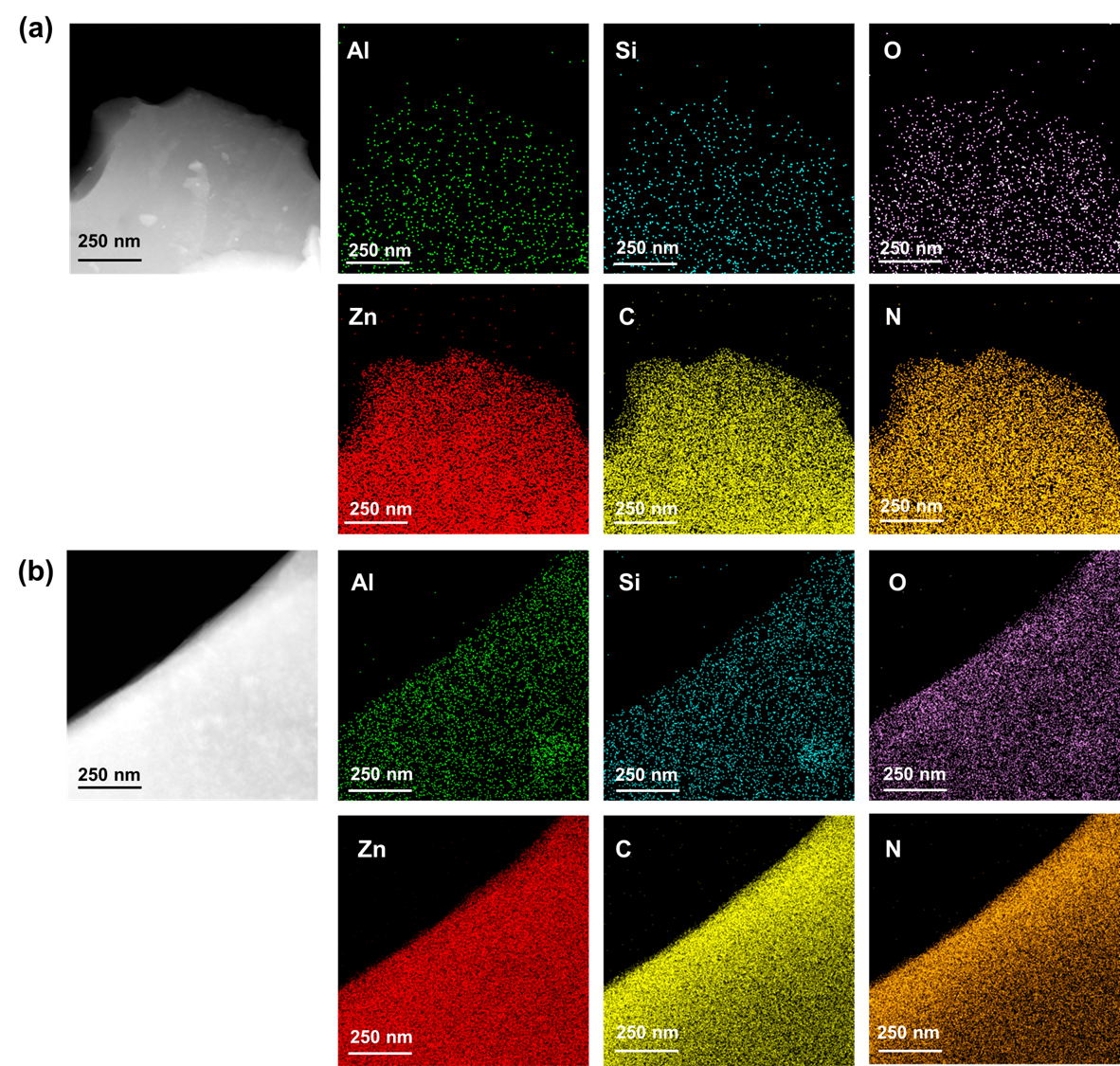
**

**Fig. S6** HR-TME-EDS mapping of zeolite glass mixed powder. (a) (a_g_ZIF-62)_0.9_(4A)_0.1_, (b) (a_g_ZIF-62)_0.7_(4A)_0.3_.

**
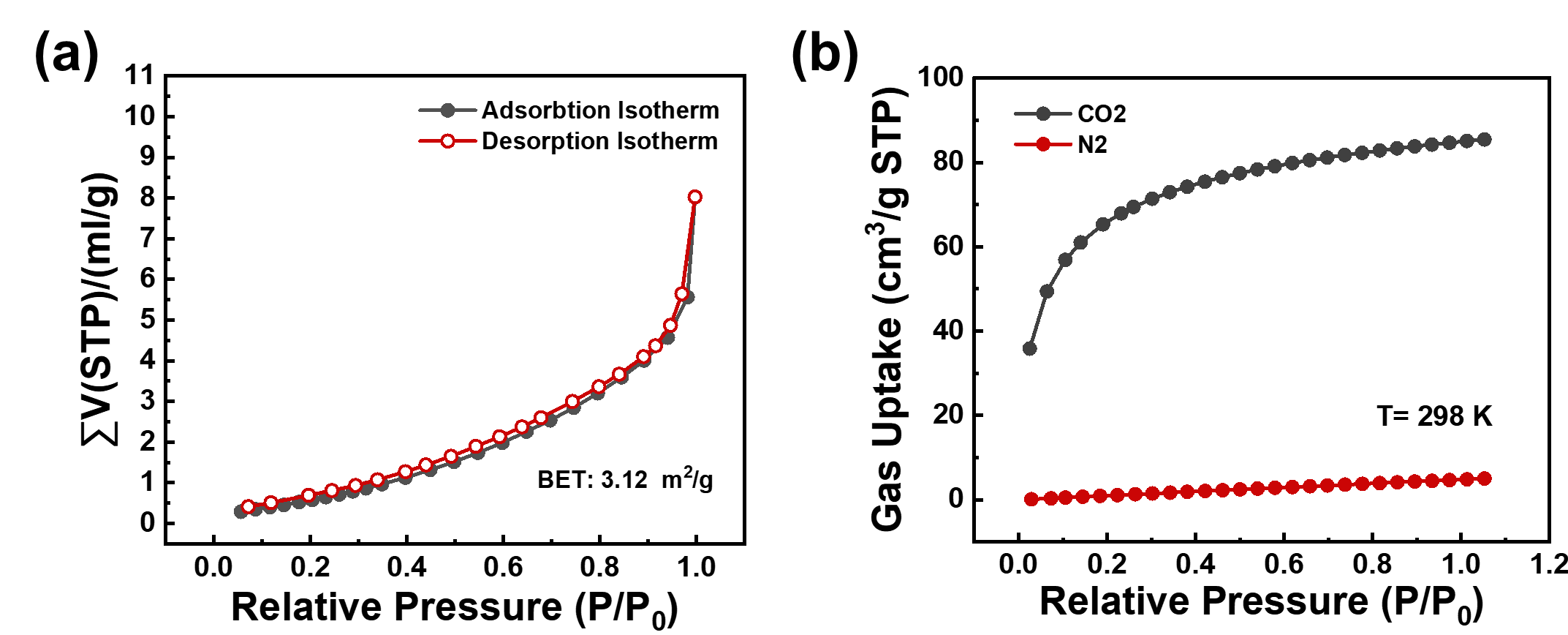
**

**Fig. S7** (a) N_2_ adsorption isotherm of 4A zeolite at 77 K. (b) Gas adsorption isotherm of CO_2_ and N_2_ on 4A zeolite at 298 K.

**
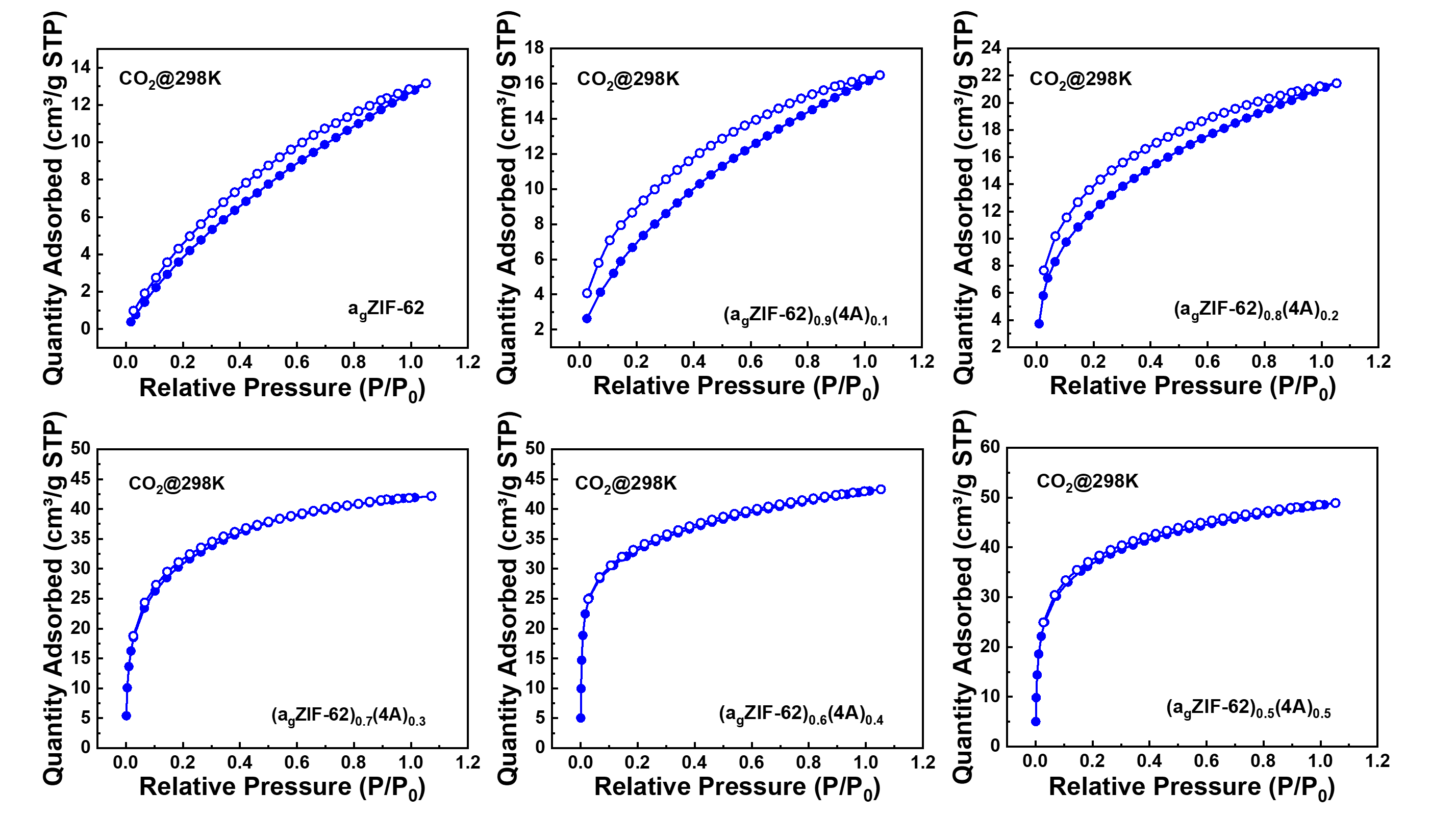
**

**Fig. S8** CO_2_ gas adsorption (solid points) and desorption (open points) isotherms of (a_g_ZIF-62)_1-x_(4A)_x_ series recorded at 298 K.

**Table S1** Elemental content of 4A zeolite

| Element | Si | Al | Na |
| --- | --- | --- | --- |
| Concentration (%) | 47.098 | 35.474 | 16.587 |

**Table S2** Comparison of pure gas separation performance between the (a_g_ZIF-62)_1-x_(4A)_x_ membranes and gas separation membranes reported in other literature.

| Membrane | Test condition  P (bar)/T (^O^C) | CO_2_ permeability (Barrer) *^a^* | CO_2_/CH_4_ selectivity | Ref. |
| --- | --- | --- | --- | --- |
| (a_g_ZIF-62)_0.9_(4A)_0.1_ | 1.5/25 | 6758 | 17.24 | This work |
| (a_g_ZIF-62)_0.8_(4A)_0.2_ | 1.5/25 | 8797 | 21.71 |  |
| (a_g_ZIF-62)_0.7_(4A)_0.3_ | 1.5/25 | 13329 | 31.73 |  |
| (a_g_ZIF-62)_0.6_(4A)_0.4_ | 1.5/25 | 17632 | 15.92 |  |
| (a_g_ZIF-62)_0.5_(4A)_0.5_ | 1.5/25 | 21253 | 6.96 |  |
| a_g_ZIF-62 | 1/25 | 2047 | 27 | [1] |
| a_g_ZIF-62/CA | 3/25 | 84.8 | 35.3 | [2] |

*^a^* 1 Barrer = 10^-10^ cm^3^ (STP) cm cm^-2^ s cmHg^-1^

**Table S3** Comparison of mixed gas separation performance between the (a_g_ZIF-62)_1-x_(4A)_x_ membranes and gas separation membranes reported in other literature.

| Membrane | Test condition  P (bar)/T (^O^C) | CO_2_ permeability (Barrer) *^a^* | CO_2_/CH_4_ selectivity | Ref. |
| --- | --- | --- | --- | --- |
| (a_g_ZIF-62)_0.9_(4A)_0.1_ | 1.5/25 | 6201 | 16.00 | This work |
| (a_g_ZIF-62)_0.8_(4A)_0.2_ | 1.5/25 | 8152 | 20.86 |  |
| (a_g_ZIF-62)_0.7_(4A)_0.3_ | 1.5/25 | 10896 | 28.81 |  |
| (a_g_ZIF-62)_0.6_(4A)_0.4_ | 1.5/25 | 16048 | 15.04 |  |
| (a_g_ZIF-62)_0.5_(4A)_0.5_ | 1.5/25 | 19958 | 6.88 |  |
| a_g_TIF-4 | 1/25 | 128 | 30 | [3] |
| a_g_ZIF-62 | 1/25 | 2602 | 36.6 | [1] |
| a_g_ZIF-62/CA | 3/25 | 45.3 | 18.1 | [2] |
| a_g_ZIF-62/PIM-30% | 2/25 | 5914 | 66.6 | [4] |

*^a^* 1 Barrer = 10^-10^ cm^3^ (STP) cm cm^-2^ s cmHg^-1^

**References**

[1] Y.H. Wang, H. Fin, Q. Ma, K. Mo, H.Z. Mao, A. Feldhoff, X.Z. Cao, Y.S. Li, F.S. Pan, Z.Y. Jiang, A MOF glass membrane for gas separation, Angew. Chem. Int. Edit., 59 (11) (2020) 4365-4369. https://doi. org/10.1002/anie.201915807.

[2] M. Mubashir, L.F. Dumée, Y.Y. Fong, N. Jusoh, J. Lukose, W.S. Chai, P.L. Show, Cellulose acetate-based membranes by interfacial engineering and integration of ZIF-62 glass nanoparticles for CO_2_ separation, J. Hazard. Mater., 415 (2021). https://doi. org/10.1016/j.jhazmat.2021.125639.

[3] H.N. Xia, H. Jin, Y.T. Zhang, H. Song, J.Q. Hu, Y. Huang, Y.S. Li, A long-lasting TIF-4 MOF glass membrane for selective CO_2_ separation, J. Membr. Sci., 655 (2022). https://doi. org/10.1016/j.memsci.2022.120611.

[4] Y. Feng, W. Yan, Z.X. Kang, X.Q. Zou, W.D. Fan, Y.J. Jiang, L.L. Fan, R.M. Wang, D.F. Sun, Thermal treatment optimization of porous MOF glass and polymer for improving gas permeability and selectivity of mixed matrix membranes, Chem. Eng. J., 465 (2023). https://doi. org/10.1016/j.cej.2023.142873.
